# Is oral and maxillofacial surgery lagging behind other specialties on X?

**DOI:** 10.1007/s10006-025-01359-x

**Published:** 2025-03-05

**Authors:** Boyu Ma, Jamie Rose, Edwin M. Rojas, Jaime Castro-Núñez

**Affiliations:** 1 Department of Oral and Maxillofacial Surgery, University of Alabama at Birmingham, Birmingham, AL USA; 2 Dental student, School of Dentistry, University of Alabama at Birmingham, Birmingham, AL USA; 3 Department of Oral and Maxillofacial Surgery, University of Alabama at Birmingham Medical Center, 1919 7th Ave S., SDB 419, 35233 Birmingham, Alabama, USA

**Keywords:** Education, Oral and maxillofacial surgery, Social media, Training, Academic productivity

## Abstract

**Purpose:**

X, formerly known as Twitter, is a popular social media platform that facilitates oral and maxillofacial surgeons to disseminate a wide array of information to a global audience. The purpose of this study is to identify the top influencers in oral and maxillofacial surgery on X, characterize who they are, and relate their social media influence to academic influence.

**Methods:**

We utilized the “BuzzSumo” Application Programming Interface to identify the most followed accounts for the topic search “oral and maxillofacial surgery.” A secondary calculator from the Mention API was used to assign a numerical score of “influence” based on their algorithm. The top 100 accounts associated with the highest engagement scores were characterized by name, status as an oral and maxillofacial surgeon/organization, location, and academic h-index.

**Results:**

We found that 52/100 (52%) of users/organizations were oral and maxillofacial surgeons/residents. There was no significant correlation between the *h-index* and the X engagement score, R^2^ = 0.8316 (*P =* 0.088). In comparison, other specialties have a higher percentage of practitioners using X and have found a correlation between h-index and X engagement score.

**Conclusion:**

Our results indicate there is a demonstrated need for the creation of more educational content from high-ranking academic sources.

## Introduction

Access to health information has allowed global communication of information between patients and practitioners. X, formerly known as Twitter, has facilitated the ability of oral and maxillofacial surgeons (OMS) to communicate with patients, educate viewers, and provide information to the public. Social media has become a tool for physicians in academia and private practice with over 6,399,146 (< 0.001%) tweets originating from 39,084 US physician accounts between 2016 and 2020 [1]. Social media influencers within the field of oral and maxillofacial surgery (OMS) benefit from interacting with a global audience to which they can dispense surgical expertise [2]. As oral health professionals produce high-quality content on social media platforms, there is a recognized need for more educational content [3].

Influencers are individuals with a high impact on social media. Successful influencers on social media can maintain user loyalty through a perceived friendship and engagement with their followers [2]. When users follow the content of their favorite social media influencers, there is a higher level of psychological well-being and loyalty [4]. Previous studies have showed that social media followers were associated with a faculty’s academic productivity [5]. As such, social media can have an impact in academic surgery [6].

In recent years, X has emerged as an instrument to progress the core values of academic surgery [6]. X can influence the number of citations in academic medicine, which factors into h-index and academic influence [7]. Increased social media citations are associated with increased dissemination and disclosure of publications [8]. The h-index is calculated by counting the number of publications for which an author has been cited by other authors at least that same number of times [7]. There is a positive correlation between citation rates and h-index amongst oral and maxillofacial surgeons [8]. Furthermore, h-index is strongly correlated with academic rank and are thresholds for promotion to associate professor, professor and endowed professor [9]. Previous studies have shown that academic oral and maxillofacial surgeons have an h-index of 6.2 ± 7.4 (range: 0–42) [3].

The authors were interested in assessing the content of the top oral and maxillofacial surgery influencers on X and their academic productivity. No studies have evaluated how X is used across specialties and compared them to oral and maxillofacial surgery influencers on social media. The investigators hypothesize that similar to other specialties, the top influencers posting OMS content are oral and maxillofacial surgeons and that their academic productivity would correlate with their social media influence. The specific aims are (1) to identify the top influencers in oral and maxillofacial surgery on X based on profession, physical location and type of posts; (2) to compare their influence on X with their academic/scientific influence on the field of oral and maxillofacial surgery; and (3) to compare OMS with other specialties.

## Methods

We utilized the “BuzzSumo” Application Programming Interface (API) from www.buzzsumo.com (Brighton, England) to identify the accounts with the highest number of followers for the topic search “oral and maxillofacial surgery.” Our inclusion criteria included organization and individual accounts that were queried using the aforementioned API. Accounts fewer than 10 total tweets, inactivity for greater than or equal to two years, or a non-publicly accessible account were excluded from the study. 185 profiles were queried until 100 influencers were reached and 85 were excluded. 100 accounts fit our inclusion criteria. Posts since 10/9/2023 were counted by two residents.

A secondary calculator from the Mention API from www.mention.com was used to assign a numerical score of “influence” based on their algorithm. The “influence” or engagement rate on X is calculated as the sum of (Likes + Retweets + Quotes + Replies) divided by the number of tweets, then by the total number of followers, and then multiplied by 100. Mention’s Twitter Engagement Calculator uses the metrics from the last 10 tweets to analyze the engagement rate. Then, the engagement index was used to reorganize the 100 profiles. The name of the individual/organization, sex, work location, and type of account were recorded. The h-index scores were recorded on 10/9/2023. The accounts with the highest followers and impact scores were ranked and sorted in Microsoft Excel.

The types of posts were also taken into account and sorted into educational, personal, marketing, and professional. Educational posts included content related to educating people about surgical techniques and oral and maxillofacial pathology. Marketing posts included content related to the promotion of the influencer’s place of work. Professional posts included content related to the promotion of professional meetings, lecture series, or recognition of the achievements of other professionals within the field. All other posts that did not easily fit into the aforementioned categories were considered personal. Statistics and graphical representations of data were performed using GraphPad Prism 10.2.0 (Boston, MA).

An online review of scientific articles was performed using Pubmed and Google Scholar with the keywords “X” or “Twitter” and “top influencer” or “academic productivity” and “social media” or “academic influence” and articles were evaluated. The inclusion criteria were studies in English, literature limited to the social media platform X, specialties relevant to medicine and surgery and relevance to our study. If a study was done at different time points, the most recent data was taken. 9 studies were identified and data was analyzed from these studies. The h-index and percentage of practitioners relative to that study were extrapolated from the data within the articles.

## Results

### Demographics of influencers

The top 100 social media influencers in oral and maxillofacial surgery are presented in Table 1. The majority (85%) of the top influencers were oral and maxillofacial surgeons. Of these, 35 were hospital/academic surgeons, 28 were residents, and 17 were private practice oral and maxillofacial surgeons. The other accounts included organizations [8], general dentist [3], pathology [1], OMS nurse [1], and OMS assistant [1]; one account was unknown based on insufficient information. Amongst all accounts run by individuals, 74 were male and 17 were female (SI Fig. 1). The average X account amongst oral and maxillofacial surgeons/organizations had 1074 followers (std. dev.: ± 1689, median: 553, range: 145 − 12,758).

**Table 1:** Influencer geographic distribution. Two influencers did not have a physical location listed

United States (20)		International (78)	
Florida	3	Saudi Arabia	21
Illinois	3	India	9
Texas	3	Pakistan	8
Virginia	3	UK	8
New York	2	Turkey	6
California	1	Jordan	3
Connecticut	1	Spain	3
Georgia	1	Egypt	3
Indiana	1	Iran	2
Kentucky	1	Venezuela	2
Washington D.C.	1	Indonesia	2
		Nigeria	1
		Colombia	1
		Kuwait	1
		China	1
		Guatemala	1
		Switzerland	1
		Qatar	1
		Netherlands	1
		Peru	1
		Syria	1
		Greece	1

**Fig. 1:**
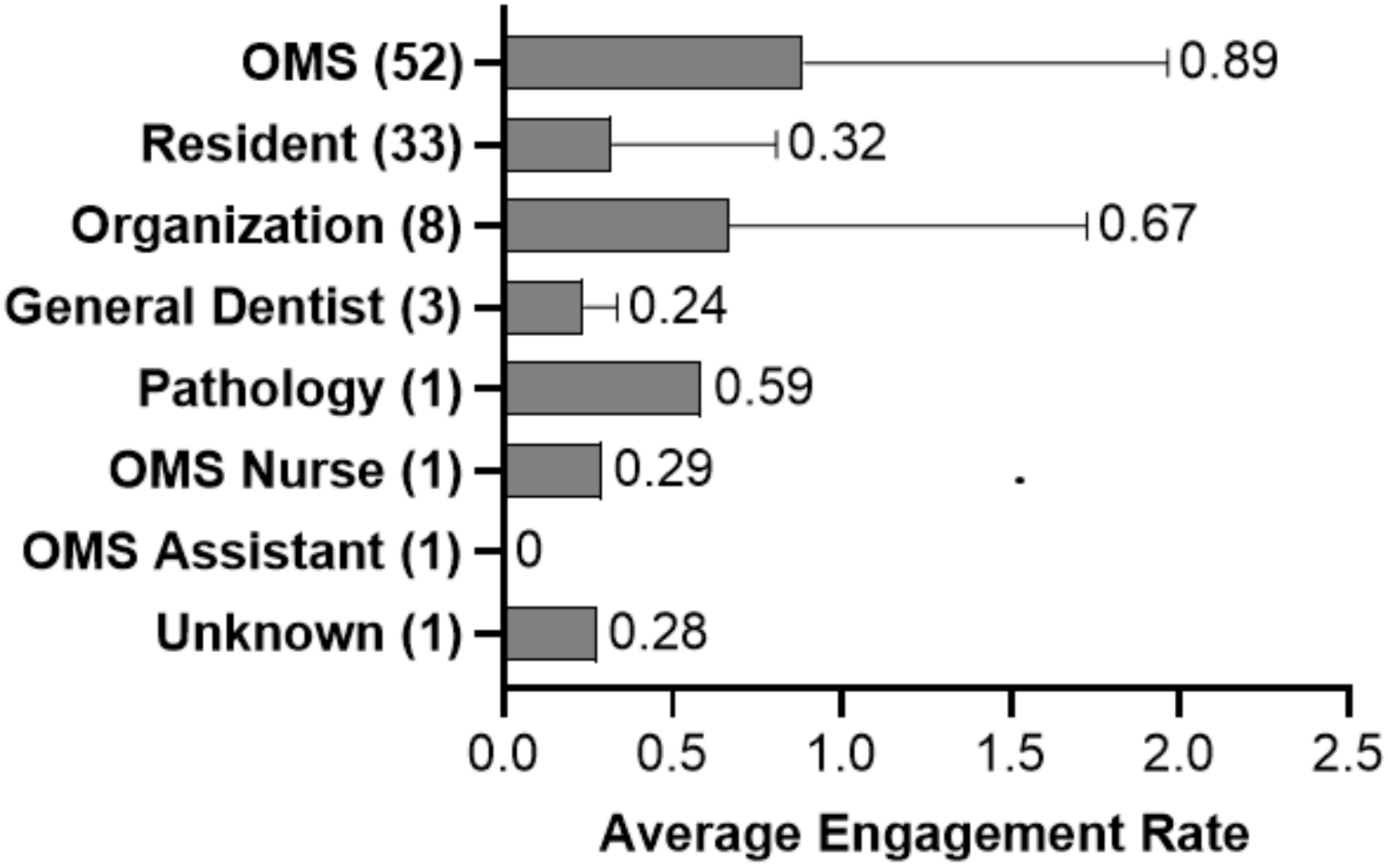
Average engagement rate per type of account by the top 100 oral and maxillofacial surgeon influencers on X. Error bars denote standard deviation (SD)

### Geographic distribution

78% of accounts were associated with users located outside of the United States. Another 20% of users were based out of the United States with 10 of the 50 states represented. Table 1 depicts the geographic distribution of X users within the United States and internationally. The top five locations in which influencers reside include Saudi Arabia [10], United States [11], India [9], Pakistan [8], and UK [8]. Within the United States, Florida, Illinois, Texas, and Virginia each had 3 influencers followed by New York [2].

### Social engagement/categorization of posts

**Fig. 2:**
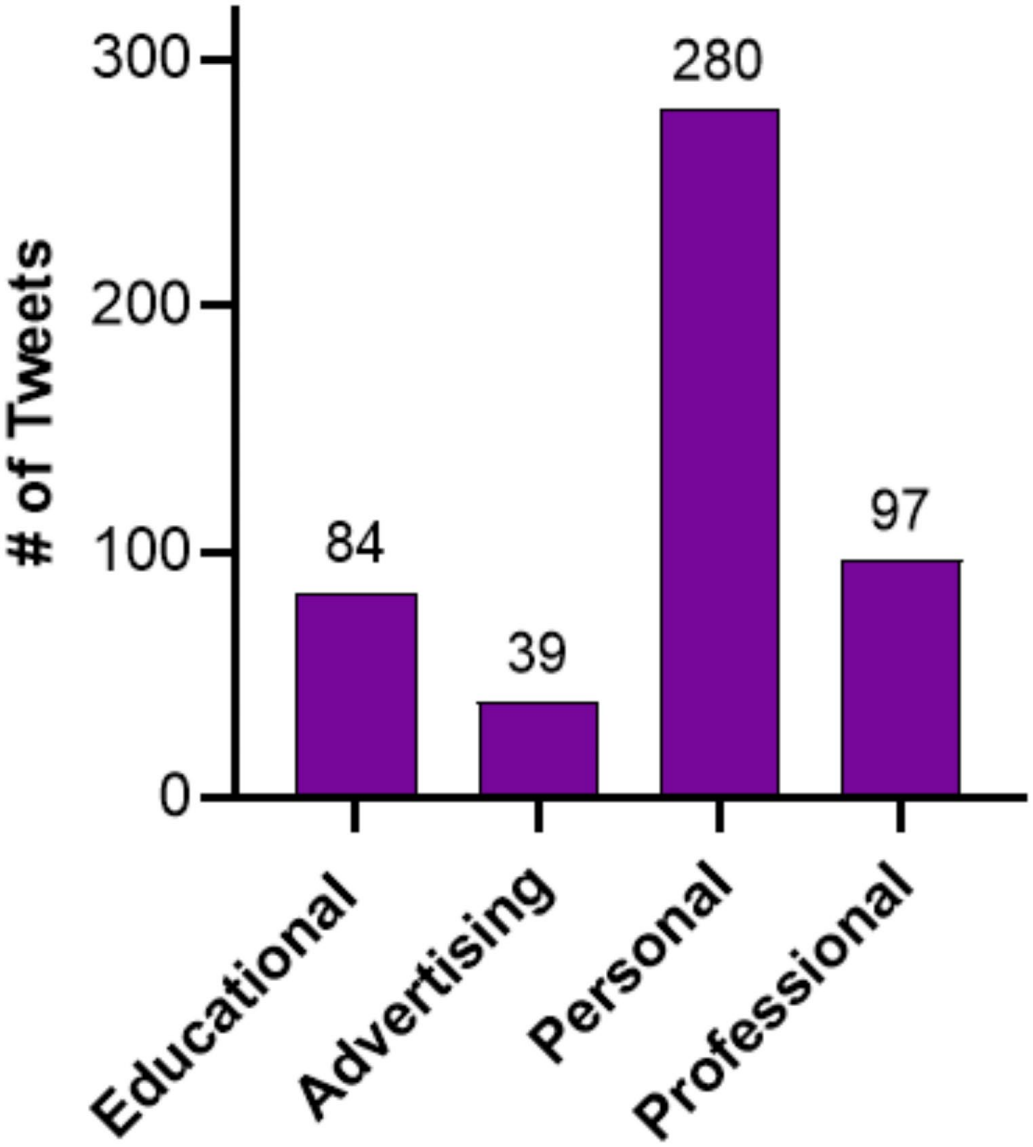
Categorization of the top 5 tweets posted by the top 100 oral and maxillofacial surgeon influencers on X

The engagement rate ranged from 0 to 5.168. There was no relationship between followers and engagement rate. The average engagement score for oral and maxillofacial surgeons who had completed all phases of training was 0.89 (range: 0.034–5.168) followed by 0.67 (0.03–3.158) for organization (Fig. 1). A total of 500 posts were categorized. There were 280 personal posts, 84 educational posts, 39 marketing posts, and 97 professional posts (Fig. 2). Across all quartiles, the personal posts were posted the most, with the 3rd quartile showing the highest proportion of personal content. Educational posts were lowest in the lowest quartile. Professional posts increase in the two highest quartiles.

### Academic productivity

**Fig. 3:**
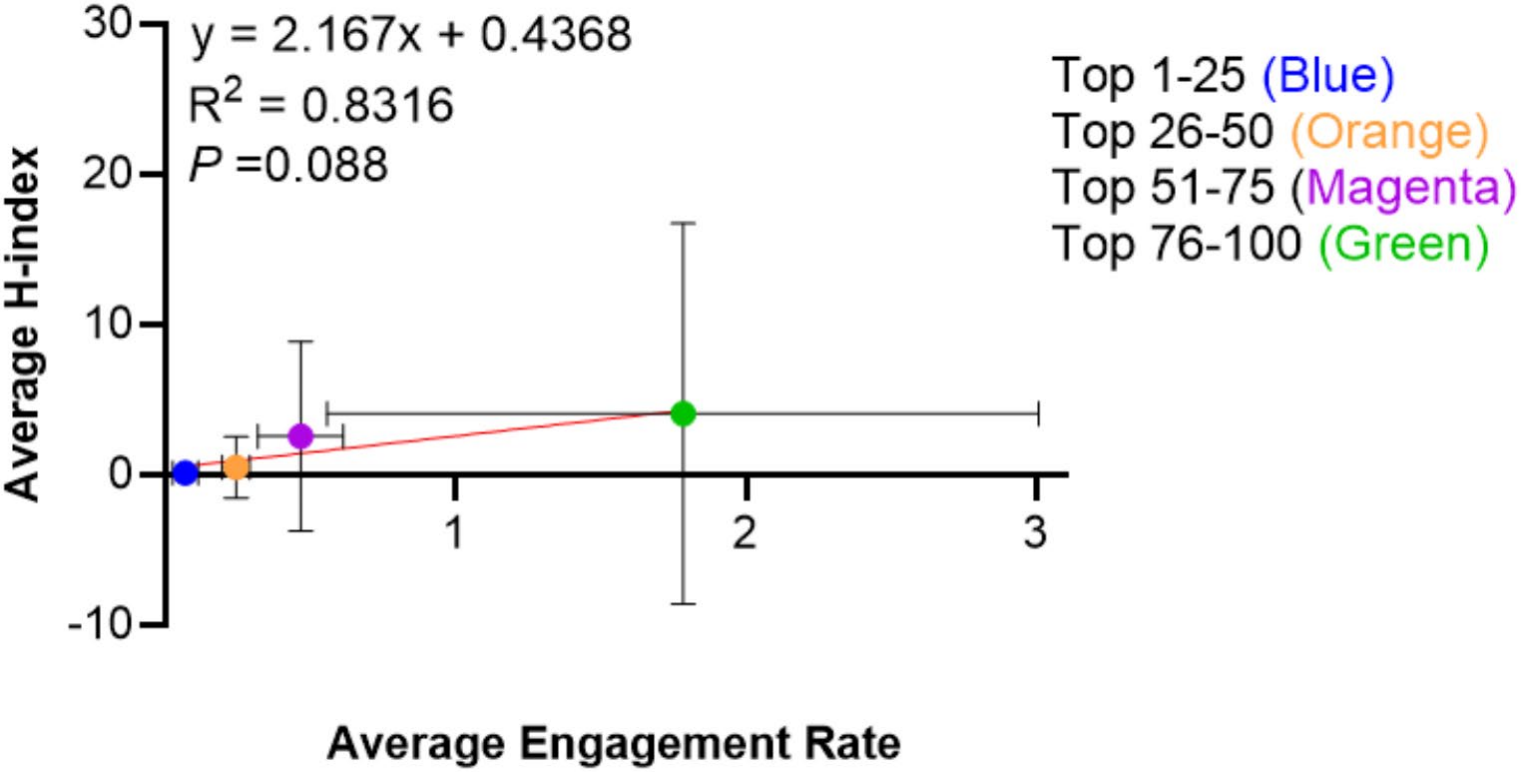
Average engagement rate vs. average h-index for top 100 oral and maxillofacial surgery X users

The average h-index of individual influencers 1–25 was 4.087 (range: 0–61; std. dev.: ± 2.643; mode: 0), 26–50 was 2.571 (0–24; ± 1.374; 0), 51–75 was 0.5 (0–10; ± 0.417; 0), 76–100 was 0.136 (0–2; ± 0.100; 0). There was no significant correlation between the h-index and the engagement score, R^2^ = 0.8316 (*P =* 0.088), as shown in Fig. 3. The organizations were excluded due to the inability to calculate the h-index.

### Comparison to other specialties

OMS had 52% of physicians among top influencers using X. Urology, cardiology and otolaryngology (ENT) were among the highest percentage of physicians using X (Table 2). The average percentage of non-OMS physicians using X was 82.1%. The compared specialties included urology (91%) [12], cardiology (88%) [13], otolaryngology (87%) [14], cardiology (88%) [15], obstetrics and gynecology (86%) [16], neurosurgery (85%) [17], orthopedic surgery (78%) [18], dermatology (77%) [19], plastic surgery (77%) [20], and general surgery (73%) [21]. OMS was 4.6 standard (< 1 percentile) deviations below the average of other specialties in terms of percentage of top influencers among their respective specialties. The average h-index for OMS was 1.5 ± 4.4. Neurosurgery top X influencers had the highest h-index with an average of 27.6 ± 19.7. The average h-index was 18.2 ± 10.7 for all specialties. There was statistically significant positive correlation of *r* = 0.6775 between percentage of physician and average H index(*P* < 0.001).

**Table 2:** Percentage of physicians within different specialties and associated h-indices. Positive correlation of (*r* = 0.6775) between percentage of physician and average h-index. (*P* < 0.001)

Specialty	Percentage of Physicians (%)	Avg. h-index (± SD)
**Urology**	**91**	**42 (16)**
**Cardiology**	**88**	**22 (32.5)**
**ENT**	**87**	**9.3 (11.0)**
**OBGYN**	**86**	**13.6 (8.5)**
**Neurosurgery**	**85**	**27.6 (19.7)**
**Orthopedic Surgery**	**78**	**13.7 (4.1)**
**Dermatology**	**77**	**13.0 (14.0)**
**Plastic Surgery**	**77**	**8.6 (10.3)**
**General Surgery**	**73**	**14.5 (8.2)**
**OMS**	**52**	**1.5 (4.4)**

## Discussion

The purpose of this study was to identify the top influencers in oral and maxillofacial surgery on X, relate their social media influence to academic influence, and compare to other specialties.

The majority of influencers (79%) identified in this study were found to be physically located outside of the United States. We found that 52% of users/organizations were oral and maxillofacial surgeons, with the majority of them located outside of the United States. This is in contrast to previous studies of social media influencers in other medical specialties which found that the majority of the top influencers resided within the United States [12–21]. For OMS, there are 0.518 oral and maxillofacial surgeons per 100,000. Social media has allowed the global reach of oral and maxillofacial surgeons on social media platforms [2]. While the United States accounts for approximately 95.4 million users on X, there exists a global network of over 500 million users on X [11].

As social media continues to grow in popularity as a means of disseminating educational content, it is ever important to characterize trends among influencers posting within the field of oral and maxillofacial surgery. Upwards of 86% of oral and maxillofacial surgeons and residents are currently active on at least one social media platform.^21^ A majority of these endorse using social media as a source of medical information [22]. There was a poor correlation between the h-index and the X engagement score, R^2^ = 0.8316 (*P =* 0.088). A majority of the content created by the top 100 influencers was personal content more so than any other content. Oral and maxillofacial surgeons use X for personal posts more so than any other type of post shown in Fig. 3. There is a relative deficiency in education posts and a demonstrated need for more educational content from high-ranking academic sources.

The average h-index amongst X influencers who had completed all phases of training was 1.52 ± 4.39, which was lower than previously reported h-index values amongst first and senior authors (7.2 and 13.7, respectively) in oral and maxillofacial surgery journals [21]. Oral and maxillofacial surgery falls behind other surgical specialties in the representation of academics within social media. A previous study reported similar h-indexes amongst the top influencers and academic surgeons in plastic surgery [19]. Social media influence has been discussed as a metric in academic promotion and tenure. A recently published article outlined the general guidelines for the academic promotion of social media [23]. As our study has indicated no significant correlation between the social engagement between the h-index and the engagement score, it is important moving forward to increase the exposure of academically productive oral and maxillofacial surgeons to the use of social media.

In comparison, this contrasts with other specialties where there was a positive correlation between social engagement and h-index. In these specialties, there are higher percentages of physicians posting and most academic influential individuals are using X compared to OMS. Why is there a disparity between OMS and other specialties? The proportion of oral and maxillofacial surgeons in academia is smaller than other fields. Urology, with the highest representation on Twitter has about 22.6% of practitioners in academia. On the other hand, for OMS, only 10% are in academia [24, 25]. Graduates of OMS are choosing narrow-scope, office based private practice jobs [26]. Academic oral and maxillofacial surgeons are currently not using X as a platform which is evident in only half of the top influencers being oral and maxillofacial surgeons and low h-index relative to the reported academic h-index of 6.2 ± 7.4 (range: 0–42) [3]. Academic oral and maxillofacial surgeons would benefit from greater academic influence as evidenced by the positive relationship between h-index and percentage of physicians using X.

The authors recognize the BuzzSumo and Mention APIs utilized for the initial query of profiles and ranking by engagement score as a limitation of this study. APIs all have different algorithms for their search engine and may yield varying results according to their ability to identify profiles based on topic searching. Some of the studies used different APIs for their study which could confound results; although most used similar APIs. On the other hand, oral and maxillofacial surgeons trained in craniofacial, head and neck or cosmetics may brand themselves differently from standard OMS. Oral and maxillofacial surgeons may choose to utilize or neglect certain terminology in their social media presence such as “oral surgery” or “facial surgery”. In addition, the number of oral health professional accounts and non-health professional accounts that utilize terms such as “oral surgery”, “facial surgery”, and “dental implantology” makes it difficult to identify influencers within the field of oral and maxillofacial surgery utilizing available APIs. Another limitation of this study stems from the ever-changing nature of social media influence. Our results merely provide a snapshot in time of the current trends in social media influence. Utilization of social media platforms and preferences change at a rapid pace. Our study only examines X, while other platforms, such as Instagram, TikTok, Facebook, and LinkedIn may have a different representation of oral and maxillofacial surgeons.

The social media platform, X, is currently under-utilized as an educational and academic platform for OMS. The top academic influencers are not using X in contrast to other specialties [12–21]. Previous studies have shown not only positive correlation between the number mentions on X and dissemination of research and knowledge, but also a positive correlation between h-index and social media influence. It is important for oral and maxillofacial surgeons to bridge the usage gap between other specialties and explore X as a platform for academic and educational benefit given its potential to disseminate information and association with academic productivity. With the large potential audience on X, oral and maxillofacial surgeons should use the platform to disseminate more information, inspire a new generation and stimulate curious minds.

## Data Availability

No datasets were generated or analysed during the current study.
